# Modifying non-coding regions of linear DNA vaccines to explore the interplay of expression and inflammation in immunogenicity

**DOI:** 10.1080/21645515.2024.2430826

**Published:** 2025-01-20

**Authors:** David C. Stirling, Maria de Miguel Ferrer, Sungwon Kim, Madina Wane, Daniel Kysh, Lisa J. Caproni, John S. Tregoning

**Affiliations:** a Department of Infectious Disease, Imperial College London, London, UK; b Touchlight Genetics Ltd, Hampton, UK

**Keywords:** Vaccine, influenza, inflammation, CpG, COVID-19

## Abstract

The COVID-19 pandemic highlighted the need for rapidly deployable, flexible vaccine platforms; particularly RNA which is now being explored for several other pathogens. DNA vaccines have potential advantages over RNA, including cost of manufacture, ease of storage and potentially lower reactogenicity. However, they have historically underperformed in large animals and human trials due to low immunogenicity. The interplay between antigen expression and the innate immune response impacts the overall immune response to DNA vaccines. Here, we explore the effect of altering non-coding 5’ regions, on the immunogenicity of a closed linear DNA platform, Doggybone™ DNA (dbDNA^TM^), produced by a rapid and scalable cell-free method. Using a mouse model, we found that enhancer sequences and DNA targeting sequences (DTS) increased influenza virus hemagglutinin (HA) expression and improved immune responses. Additional CpG motifs did not provide any immune benefit. We also found that the effect of non-coding sequences was target specific, with differing effects in influenza HA, SARS-CoV-2 Spike and eGFP constructs. To separate the effects of immune sensing of the DNA construct and the expression of the encoded antigen, we combined a separate CpG oligodeoxynucleotide (ODN) with the highest expressing DNA vaccine; we observed reduced expression, but higher inflammation resulting in equivalent immunogenicity. Further refinement is required to fully understand the interplay of factors required for the induction of protective immunity by DNA vaccines.

## Graphical Abstract

**Figure:**
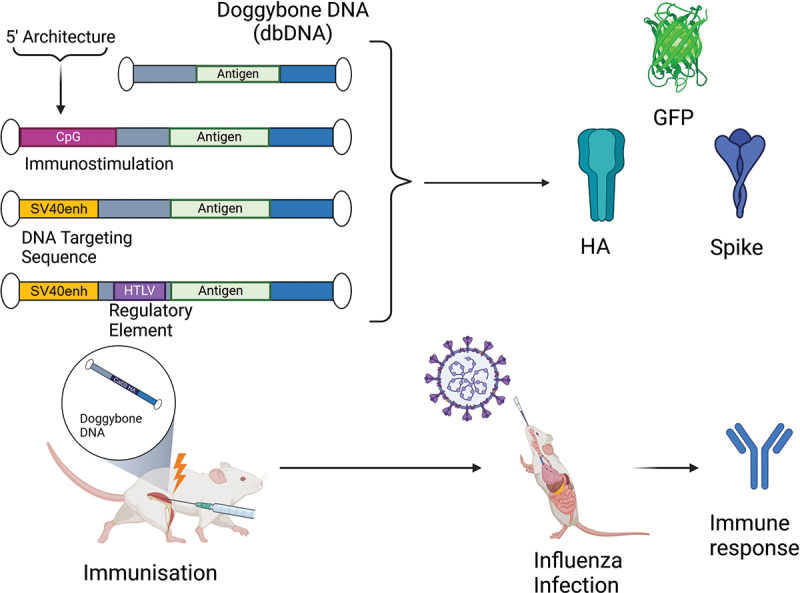

## Introduction

Following the COVID-19 pandemic and the success of RNA vaccines, there is renewed interest in the development of the other nucleic acid vaccine platform, DNA. Synthetic DNA vaccines share many of the attractive properties of RNA vaccines: they require only nucleotides and knowledge of a target’s genetic sequence for construction and they are a highly flexible and safe platform for new vaccine development. Additionally, as nucleic acid vaccines are made of the same raw materials, single manufacturing lines can be rapidly adapted and scaled to respond to emergent threats, giving the platform heightened utility in pandemic preparedness.^1^ However, issues with economic and equitable scale up of RNA have prompted a reevaluation of the DNA because of its cheaper cost, greater stability and ease of manufacture. In the previous decades of development, DNA vaccines have been shown to be immunogenic and protective against multiple pathogens including influenza and cancer in various model organisms.^2,3^

Conventional DNA vaccines are made of plasmids containing an expression cassette that encodes the protein target, but also necessarily include additional backbone sequences to support the plasmid propagation in bacterial cultures. Whilst a relatively simple process, utilizing bacterial fermentation of *Escherichia coli* in production necessitates several clean-up and safety checks before the release of a GMP product and can slow vaccine production;^3^ in addition GMP cell banks are required which can add further cost and time constraints. Doggybones™, or doggybone DNA (dbDNA™), are minimal closed linear DNA constructs that are produced using an *in vitro* enzymatic amplification process that results in a covalently closed linear DNA construct, absent of the extraneous sequences common to pDNA (bacterial backbone). As a result, dbDNA™ can be amplified to GMP at scale with a much simpler manufacturing process and smaller footprint than plasmid DNA. In head-to-head comparisons in small animal models, the immunogenicity of dbDNA™ has been shown to be comparable to or better than plasmid DNA.^4,5^

Whilst very recent phase 1/2 studies have indicated that a personalized neoantigen plasmid DNA cancer vaccine delivered with electroporation was immunogenic in 86.4% of participants,^6^ further optimization of DNA vaccines is required to overcome hurdles in successfully translating DNA vaccines to humans, where they have previously displayed lower immunogenicity.^7^ Several approaches have been explored to increase DNA vaccine immunogenicity, including changes to genetic sequence, the use of adjuvants, and alternative delivery routes or methods.^7,8^ The prevailing dogma is that increasing the level of antigen expression from a DNA vaccine will lead to higher and more protective immune responses. This can be achieved by increasing vaccine dosage, but there are caps on dose relating to manufacturing costs and reactogenicity especially if formulated. Therefore, ongoing research aims to increase immunogenicity whilst being DNA dose sparing.^9^ Supporting transgene translation with the use of powerful promoters and enhancers such as the CMV promoter or the upstream region of the NTC7382 plasmid, here called triple enhancer (TEnh), are examples where modification of the regulatory architecture of plasmid vaccines has been used to improve vaccine function by increasing protein expression.^10,11^ An important factor that limits DNA vaccine translation is the need to cross the nuclear envelope, which acts as a highly restrictive barrier in cells not undergoing mitosis.^12,13^ By including DNA targeting sequences (DTS) that associate with proteins that routinely cross the nuclear envelope, plasmid nuclear entry can be significantly increased.^14^ The SV40 enhancer is a known DTS, containing binding sites for the transcription factors AP1, AP2, AP3 and NF κB that are ubiquitously expressed in human cells.^15^ Including (or adding) the SV40 enhancer in plasmid sequences has been shown to promote DNA nuclear localization and increase protein expression in mouse muscle tissue 20-fold.^16,17^

Another factor to consider in the formation of immune memory is the stimulation of an acute innate immune response. Foreign DNA is inherently inflammatory, recognized by a diverse array of pattern recognition receptors (PRRs).^18,19^ The PRRs engaged depend on the sequence and shape of the DNA molecule; therefore, the response to DNA vaccines can be changed by modifying the DNA sequence. Unmethylated cytosine-phosphate-guanosine CpG motifs are recognized by the PRR Toll-like receptor 9 (TLR9), and they have been shown to enhance cellular responses in mice and alter responses from human cells.^20,21^ Different CpG sequences have been found to have different immune effects, with effects also being influenced by delivery as oligodeoxynucleotide (ODN) or as part of a longer DNA molecule.^22^ The DNA sequence, 5’ TCGTCGAACGTTCGAGATGAT 3’, is a CpG motif derived from the CpG-rich ODN C274 that activates plasmacytoid DCs (pDCs) and stimulates B cell responses in human PBMC.^23,24^

The inclusion of the C274 sequence in vaccine constructs has been associated with improved protection of mice from respiratory syncytial virus (RSV) challenge.^25^ Increasing the inflammation following immunization is likely beneficial to the vaccine response in many cases by increasing antigen presentation and immune cell activation. However, higher levels of innate response to nucleic acid may also trigger antiviral mechanisms or CTLs to eliminate antigen expression, resulting in an overall weaker vaccine response.^26^ Higher inflammation may also have consequences in terms of reactogenicity. The optimal balance of protein expression and innate immunity for DNA vaccines, and the sequences to support them are an open question that further study is required to elucidate.

The aim of the current study was to compare the immune response elicited by dbDNA™ vaccines with non-coding sequences designed to increase protein expression or stimulate innate immunity to better inform future vaccine design. We compared the immunogenicity of dbDNA^TM^ vaccines in the presence and absence of DTS or CpG motif in a mouse model during an influenza challenge. We discovered trends in the data indicating that the SV40 DTS enhances the expression of HA, but not other model proteins. DTS also supported an increase in anti-HA IgG levels, which correlated with reduced disease. These studies show that altering non-coding regions of DNA vaccines can affect the immune response.

## Materials and methods

### Oligodeoxynucleotide (ODN)

ODN C274 (5’-TCGTCGAACGTTCGAGATGAT-3’) and Con274 (5’-TGCTGCAAGCTTGCAGATGAT-3’) were sourced commercially from Eurofins Genomics, and each was synthesized with phosphorothioate backbone.

### Doggybone^TM^ (dbDNA™) construction

The codon-optimized amino acid sequences of influenza A virus (H1N1) strain (A/California/07/2009) hemagglutinin (HA), SARS-CoV-2 Spike and eGFP were synthesized and cloned into proTLx-K (standard expression cassette with CMV enhancer/promoter and SV40 late polyadenylation signal) with and without TEnh located between CMV promoter and the genes of interest (GOI) ORF. The db SARS-CoV-2 Spike sequences originated from the SARS-CoV-2 Wuhan Spike open reading frame, with two amino acids substituting at K986P and V987P for stabilization. To generate CpG motif and DTS version, the CpG-rich region (containing 5 repeats of 5’ TCGTCGAACGTTCGAGATGAT 3’) and SV40 enhancer sequence (containing two tandem 72bp DTS sequences), respectively, are inserted into the upstream of the CMV enhancer/promoter into standard and TEnh version of plasmids.^16^ The resulting plasmid templates contain the expression cassette flanked by *E. coli* phage N15 TelN protelomerase binding sites. dbDNA™ was produced as previously described.^5^ Briefly, the template plasmid was denatured using NaOH and then quenched in the reaction buffer containing custom primers, dNTPs, Phi29 polymerase and pyrophosphatase. Upon mixing, the reaction was incubated at 30°C for 30 h or 72 h. Concatemeric DNA was processed by the addition of TelN protelomerase, restriction enzyme, exonucleases. The digest mixture was cleaned from reaction components and precipitated using polyethylene glycol (PEG) 8000. DNA Concentration determined by the absorbance method 260 nm with acceptance criteria > 1.0 mg/mL.

### Cell culture and transfection

HEK293T (from ATCC) were cultured in Dulbecco’s modified Eagle’s medium (DMEM) (Gibco) supplemented with 10% fetal bovine serum (FBS) (Sigma Aldrich) and 2 mM L-Glutamine (Gibco). Human skeletal muscle cells (HSMCs) were gifted by Dr Rachel Barton. HSMCs were grown in Skeletal Muscle Cell Growth Medium (PromoCell) with supplement mix. All cells were regularly tested for mycoplasma contamination and used for *in vitro* transfection study at passage numbers less than 30. Cells were grown to 80% confluency in 24-well cell culture plates. 200 ng and 300 ng of dbDNA^TM^ were transfected into HEK293T cells and HSMC cells, respectively, using the PEIpro transfection reagent at a 1:4 dbDNA-to-PEIpro ratio. Cells were incubated at 37°C 5% CO2 for 24 h in a Culture Safe CO2 Precision 190D (LEEC).

### Flow cytometry of cultured cells

At the 24 h post-transfection (pt), the cells were gently washed with PBS and then detached by incubation with TrypLE™ Express Enzyme (Gibco) and gentle rocking. The reaction was quenched with 10% FBS in PBS and cells were transferred to 96-well U-bottom plates. Cells were pelleted by centrifugation at 400 xg for 5 min. Cells were washed by resuspension in 150 µL of PBS and centrifugation at 400 xg for 5 min. Cells were resuspended in 100 µL of LIVE/DEAD™ Fixable Violet (Thermofisher L34955) at a 1:400 dilution and incubated in the dark for 30 min at 4°C. After the cells were washed and blocked with 2% FBS for 1 h at 4°C, they were incubated with primary antibody in PBS (1 in 200 Influenza A Virus Hemagglutinin (C102) (SANTA CRUZ BIOTECHNOLOGY, INC), or 1 in 2000 Anti SARS spike glycoprotein antibody [1A9] (abcam)) and a secondary antibody (Goat Anti Mouse IgG PE/Cy5.5® (abcam), 1 in 12,000 in 50 µL of PBS) sequentially for 1 h at 4°C. After fixation with 2% PFA for 20 min at 4°C, the cells were resuspended in PBS for analysis by flow cytometry. The positive fraction of cells was identified, and the MFI of the positive fraction was calculated using FlowJo analysis software. The MFI values were normalized by dividing by the mean MFI of the Family1 (F1) group.

### Murine vaccine challenge

Eight to twelve week-old female BALB/c mice were obtained from Charles River Ltd (Edinburgh, UK) and kept in specific-pathogen-free (SPF) conditions in accordance with the United Kingdom’s Home Office guidelines and animal care procedures. All experiments were performed in the SPF room in the animal facility on a 12-h light/dark cycle at 20–24°C with 55% ± 10% humidity at Imperial College London, St Mary’s Hospital Campus. All work was approved by the Animal Welfare and Ethical Review board at Imperial College London, and studies were in accordance with the Animal Research: Reporting of In vivo Experiments (ARRIVE) guidelines.

Mice were anaesthetised prior to immunizations or intranasal infections by inhalation of 2% isoflurane carried by oxygen. Anaesthetised mice were injected intramuscularly (i.m.) into the right anterior tibialis with dbDNA™ in 50 µL of PBS, followed by electroporation (EP) of the injection site. Electroporation was carried out using a CUY21 EDIT system (BEX, Japan), delivering 10 pulses of 150 V with switched polarity between pulses. Virus was diluted to a desired PFU in sterile PBS, and 100 µL of diluted virus was delivered intranasally with a Gilson pipette. The weights of mice were measured daily using an electronic balance to monitor disease progression. The humane cutoff for weight loss was set at 80%. Mice terminated for humane reasons were euthanized by cervical dislocation.

### Tissue sampling

Blood sera were sampled by mice at specific time points after immunization by venepuncture of the tail or at the endpoint by arterial bleed. Sera was separated from blood after clotting by centrifugation at 35,514 xg for 15 min in a Mikro 220 R micro-centrifuge (Hettich). Mice were euthanized by intraperitoneal (i.p.) injection with pentobarbitone (20 mg dose, Pentoject, Animalcare Ltd. UK). The left lobe of the mouse lung was removed and snap-frozen at −80°C for viral load analysis. Spleens were extracted, and splenocytes were separated by mechanical dissociation of spleen tissue through a 75 µm cell strainer. Cells were pelleted by centrifugation at 929 x*g* and then resuspended and incubated in 1 mL of ACK lysis buffer for 5 min at room temperature. The reaction was halted by addition of RPMI and then the cells were pelleted again and resuspended in RPMI 1640 medium with 10% FBS. Viable cell numbers were determined by trypan blue exclusion.

### ELISA

Antibodies specific to influenza H1N1 virus were measured in sera using a semiquantitative ELISA. MaxiSorp 96-well plates (Nunc) were coated with antigen at 1 µg/mL of HA (Sino Biological) or Recombinant SARS-CoV-2 Spike His Protein, CF (R&D). Standard wells were coated with 50 µL of IgG α-mouse κ and α-mouse λ antibodies each at a concentration of 0.4 mg/mL (Novus Biologicals). Plates were covered and incubated at 4°C overnight. Plates were blocked with 1% BSA in PBS. Immunization sera or a serially diluted matching antibody subtype of known concentration (Southern Biotech) as a standard to quantify specific antibodies was added to wells followed by subtype specific HRP-conjugated goat anti-mouse antibody (Abcam). TMB (Thermo Fisher) with 2 N H_2_SO_4_ as a stop solution was used to detect the response and absorbance read at 450 nm using a FLUOstar® Omega Plate Reader. Cytokine ELISAs were performed using R&D kits according to the manufacturer’s instructions with half volumes.

### Hemagglutination inhibition (HAI) assay

Serum samples underwent pretreatment with Receptor Destroying Enzyme (RDE, Denka Seiken) for 18 h at 37°C, followed by enzyme inactivation at 56°C for 1 h. The RDE-treated serum was then serially diluted in a 2-fold manner across a V bottom plate (Merck) using 1X PBS and allowed to incubate with a pre-diluted solution of 4 hemagglutinating units of A/California/07/2009 (H1N1) virus per well for 30 min at room temperature. 50 μL of 1% turkey erythrocytes (Envigo) diluted in PBS was added to each well for the virus to agglutinate. The plate was then incubated for another 30 min at 4°C before the result was recorded.

### Flow cytometry

Live lung cells and cells from BAL were plated out onto a U-shaped 96 well plate then spun down at 929 xg for 2 mins at 4°C. 100 µl of Live/Dead violet dye (ArCTM, Catlog: A10346) was added for 20 mins at 4°C in the dark, the plate was then centrifuged at 929 xg for 2 mins and the supernatant taken off. The cell pellet was resuspended in Fc block (Clone: 2.4G2) in PBS-1% BSA and stained with the following surface antibodies: FITC anti-mouse CD3 (Clone: 12A2, Cat: 100204, BioLegend), APC-H7 anti-CD8 (Clone: 53–6.7, Cat: 560247 BD Biosciences), PerCP-Cy5.5 anti-mouse CD4 (Clone: RM4-5, Cat: 100540 BioLegend), BV711 anti-mouse CD44 (Clone: IM7, Cat: 103057 BioLegend), PE-Cy7 anti-mouse CD62L (Clone: MEL-14, Cat: 104418 BioLegend) for one hour in the dark. Excess antibodies were washed off with 1% BSA in PBS three times before filtered through the FAC tubes on an LSR Fortessa Flow cytometer (BD) and FlowJo. Fluorescent minus one (FMO) controls were used for surface stains.

### ELISpot of splenocytes

ELISpot assays were conducted using a commercially available kit from Abcam (ab64029), according to the manufacturer’s guidelines. The cells were stimulated with 1.25 μg/mL of anti-CD28 (Clone 37.51, BD) and pre-constructed 15 mer sequences with 11 amino acids overlap peptides derived from influenza A (H1N1) HA (Peptivator, Miltenyi Biotech) or SARS-CoV-2 (Peptivator, Miltenyi Biotech). The spots were enumerated utilizing the AID iSpot reader in conjunction with the ELISpot Reader software V 7.0.

### Influenza viral load

Frozen lung tissue was disrupted by pulverization by stainless steel ball bearings in Trizol using a TissueLyzer (Qiagen). RNA was converted into cDNA using GoScript™ Reverse Transcriptase (Promega), Quantitative PCR was carried out using 0.1 μM forward primer (5ʹ-AAGACAAGACCAATYCTGTCACCTCT-3ʹ), 0.1 μM reverse primer (5ʹ-TCTACGYTGCAGTCCYCGCT-3ʹ), and 0.2 μM probe (5ʹ-FAM-TYACGCTCACCGTGCCCAGTG-TAMRA-3ʹ) for the influenza M gene on a Stratagene Mx3005p (Agilent technologies).^27^ M-specific RNA copy number was determined by a standard curve matching to a influenza M gene-encoded plasmid.

### Multiplex cytokine assay

Blood cytokine levels were measured using commercial multi-spot U-PLEX kits from Meso Scale Discovery (MSD) and performed according to the manufacturer’s instructions. Data was analyzed, and lower limits of quantification (LLOQ) were determined using MSD DISCOVERY WORKBENCH software.

### Statistical analysis

Statistical analyses were performed using GraphPad Prism 10 (GraphPad Software Inc., La Jolla, CA, USA), with an alpha significance level of 0.05. Multiple correlations were performed by Pearson r, while individual correlations were performed by simple linear regression.

## Results

### Design of linear DNA vaccine constructs with altered non-coding regions

**Figure 1.**
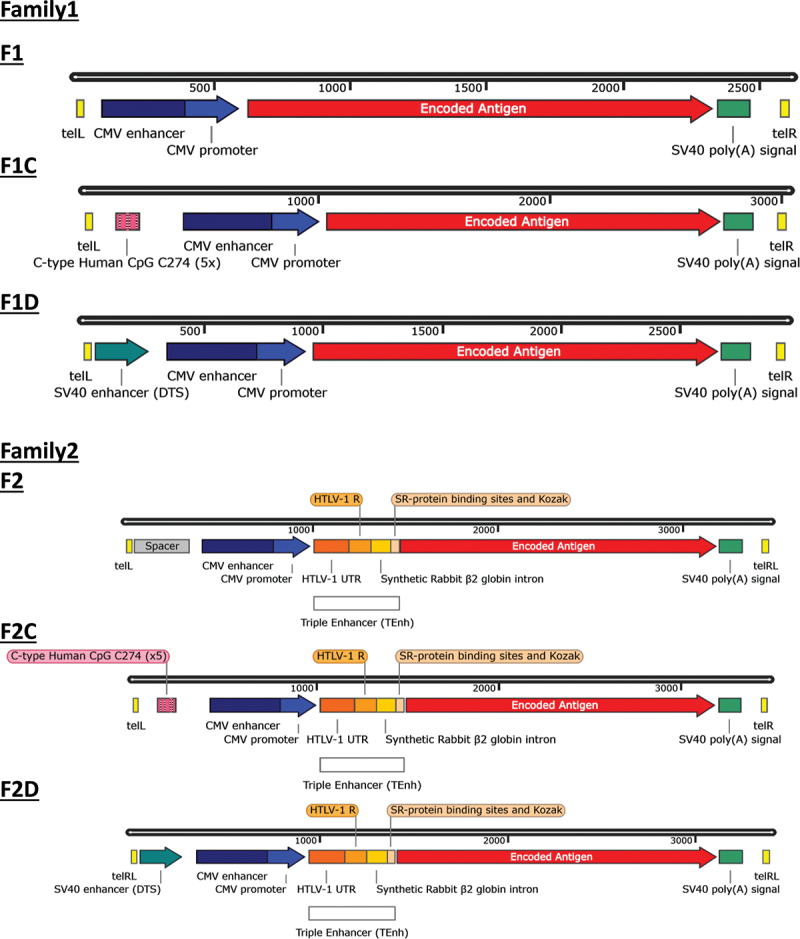
Schematic of the dbDNA™ architectures studied. All dbDNA™ included the CMV enhancer and promoter and an SV40 poly(A) signal. dbDNA™ in Family2 included a triple enhancer (TEnh). F1C and F2C include a CpG rich region, and F1D and F2D include the SV40 enhancer sequence.

We first designed dbDNA™ vaccines with different sequence modifications, which were hypothesized to affect expression or innate immune sensing (Figure 1). Two core designs were used: Family1 (F1) and Family2 (F2). F1 is comprised of the CMV promoter and enhancer, a multiple cloning site where GOI are inserted, and SV40 late poly(A) signal. F2 has the same basic features as F1, but includes additional elements between CMV enhancer/promoter and GOI to enhance protein expression. These additional elements are the regulatory R region from the 5′ long terminal repeat (LTR) of human T-cell lymphotrophic virus type 1 (HTLV-1), an SR protein binding sequence, and synthetic rabbit β-globin intron, referred to here as triple enhancer (TEnh). The TEnh has been shown to improve protein expression and immune responses to DNA vaccines in mammals.^28,11^ Vaccines with altered sequences were made in each dbDNA™ Family with either a CpG-rich vaccine (F1C/ F2C) or a DTS derived from the SV40 enhancer (F1D/ F2D). The supporting sequences were appended to F1 upstream of the CMV promoter site or substituted in place of the upstream spacer in F2.

### Effect of modifying dbDNA™ on transfection efficiency is influenced by structure, antigen and cell type

**Figure 2.**
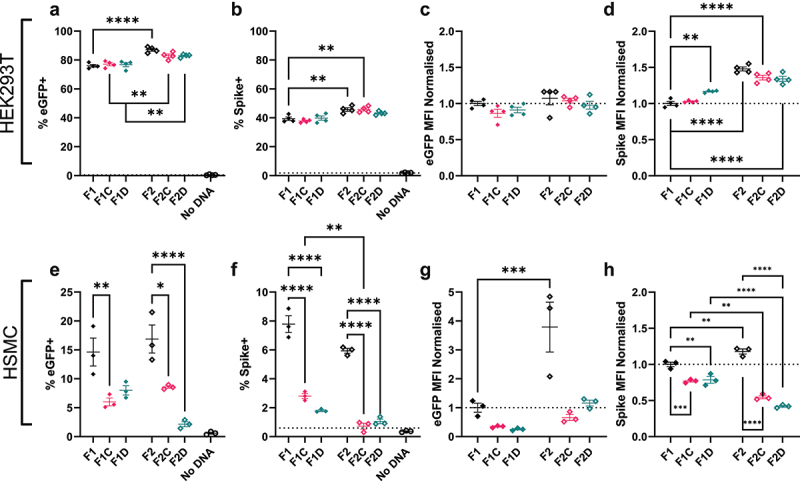
Transfection efficiency differs between model proteins. Cells were grown to 80% confluency on cell culture plates and then transfected with dbDNA™ expressing eGFP or SARS-CoV-2 Spike protein using the PEIpro® transfection system. HEK cells were transfected with 200ng DNA, HSMc cells with 300ng. Percentage of total cells expressing transfected protein was measured by flow cytometry at 24 hours after transfection and the median fluorescence intensity (MFI) within the positive fraction of cells was calculated using FlowJo analysis software. Points represent individual tests; lines represent mean of n ≥ 3 tests ± SEM * p < .05, calculated using ANOVA and posttest.

We first wanted to investigate the effects of dbDNA™ sequence modification on the efficiency of protein expression. dbDNA™ of each design encoding eGFP protein or the SARS-CoV-2 Spike protein were transfected into cells *in vitro* with the PEIpro™ transfection system, and the resulting protein expression efficiency and magnitude were measured 24 hours after transfection by flow cytometry. A significantly greater proportion of cells had detectable expression of eGFP from F2 constructs than F1 constructs (p < .0001), Figure 2a. A similar significant increase was seen in SARS-CoV-2 Spike protein expression for the F2 (p < .01) and F2C (p < .05) constructs than their F1 equivalent (Figure 2b). A subtly different pattern to percentage positive was seen for the amount of expression within positive cells (determined by mean fluorescence intensity: MFI); none of the modifications affected GFP MFI (Figure 2c). However, increased MFI was observed following transfection with F2 constructs encoding Spike dbDNA™ (Figure 2d). HEK293Ts are optimized for DNA expression due to SV40T antigen expression and lack innate DNA responses; to explore possible interplay with the cell intrinsic immune system, expression following dbDNA™ transfection was explored in another cell type.^29^ The same experiment was performed transfecting immortalized human skeletal muscle cells (HSMCs). In this system, the proportion of eGFP^+^ (Figure 2e) or Spike^+^ (Figure 2F) cells was significantly higher in the unaltered F1 or F2 configurations. A similar effect was seen with MFI, the inclusion of CpG-rich regions or DTS reduced the MFI of eGFP (Figure 2g) or Spike (Figure 2h). These results indicate that altering DNA architecture can impact expression, but the effects are both cell line and antigen dependent.

### Modifying dbDNA™ architecture has no effect on the immunogenicity of SARS-CoV-2 spike vaccination

**Figure 3.**
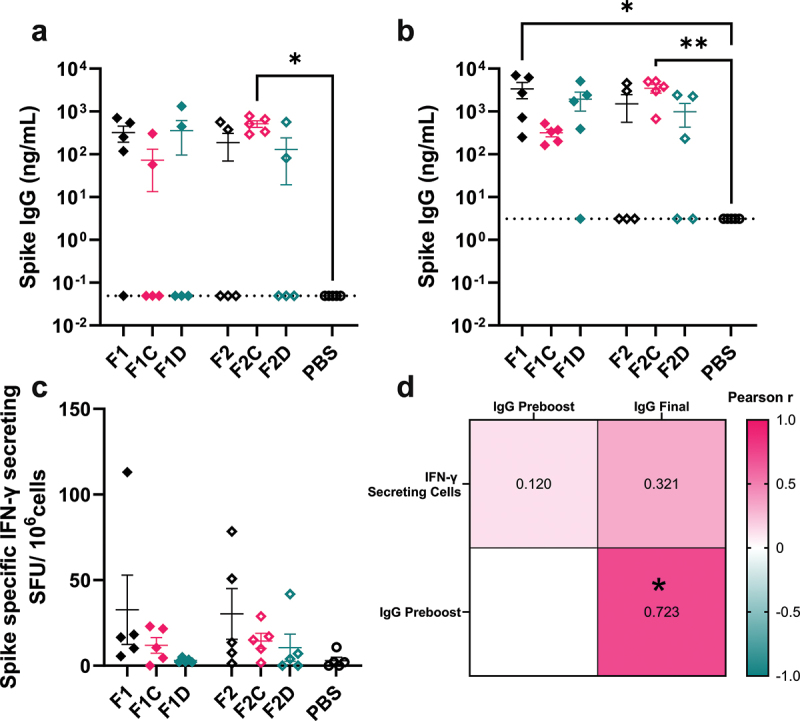
Support sequences do not affect immunogenicity of SARS-CoV-2 spike encoding dbDNA™ vaccine. Female BALB/c mice aged 8–12 weeks were vaccinated with 10 µg Spike encoding dbDNA™ in the right anterior tibialis muscle with electroporation and a second identical vaccination at 4 weeks. Blood was collected to measure anti Spike antibody responses at 4 (a) and 6 weeks (b) by tail bleed. Mice were sacrificed 6 weeks after initial immunization and spleens were collected to measure spike specific cell responses by ELISpot (c). Pearson correlation r values were computed between IgG titers and IFNγ cell counts (d). Points represent individual mice; lines represent mean of n ≥ 5 mice ± SEM; this study was performed once. * p < .05, ** p < .01, calculated using ANOVA and posttest.

Having seen some impact on *in vitro* expression, we explored the impact of the CpG-rich region or the DTS on the immune response to dbDNA™. Mice were immunized with 10 µg of Spike encoding dbDNA™. This was performed as a prime-boost with 4 weeks between injections; 2 weeks after the booster immunization, mice were euthanized, and blood and tissue samples were collected for immunogenicity analysis. Sero-conversion was variable after first dose, and only mice immunized with F2C had significantly higher SARS-CoV-2 Spike-specific IgG levels than the PBS control at 4 weeks (p < .05; Figure 3a). A similar pattern of antibody titers persisted at 6 weeks (Figure 3b), with a notable increase in titer in the F1 group. Specific T-cell responses to vaccination evaluated at 6 weeks by ELISpot analysis of splenocytes revealed modest responses; a trend emerged indicating greater T-cell responses following vaccination without supporting sequences, followed by vaccination with the CpG-rich region, and lastly, vaccination with DTS (Figure 3c). Antibody responses to immunization did not correlate with T-cell responses (Figure 3d). These data suggests that changes in dbDNA^TM^ had little effect on immunogenicity with constructs encoding spike antigen.

### F2 architecture and DTS enhance expression of dbDNA™ encoded influenza antigen in vitro

**Figure 4.**
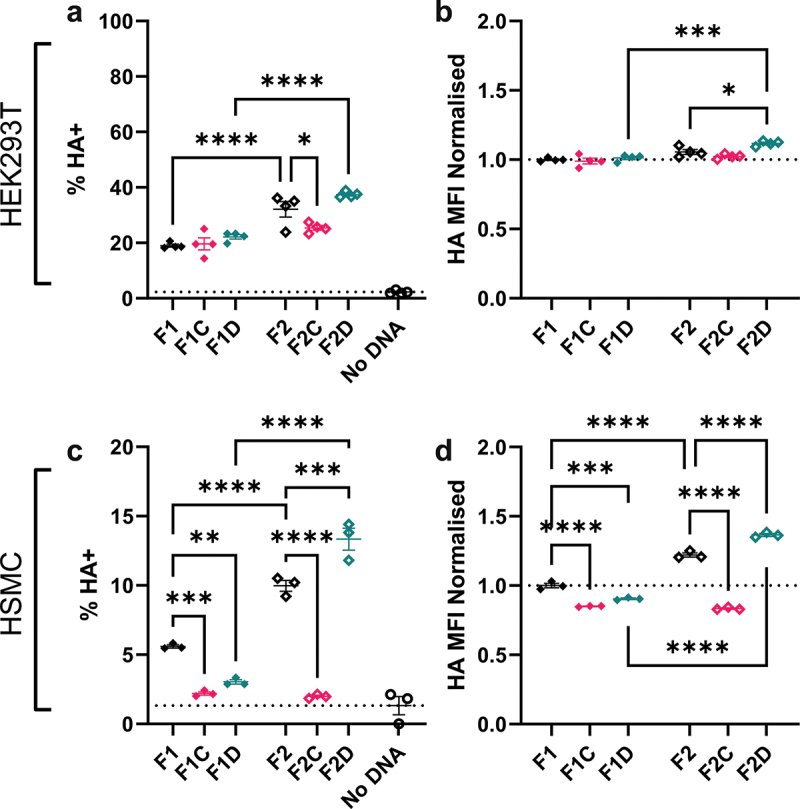
Family2 Triple Enhancer and NIS support sequence increase HA expression in vitro. Cells were grown to 80% confluency on cell culture plates and then transfected with dbDNA™ expressing Cal09 Flu HA protein using the PEIpro® transfection system. HEK cells were transfected with 200ng DNA, HSMc cells with 300ng. Percentage of total cells expressing transfected protein was measured by flow cytometry at 24 h after transfection and median fluorescence intensity (MFI) within the positive fraction of cells was calculated using FlowJo analysis software. HEK293T cells were transfected with 200ng dbDNA in 24 well plates (a, b) and HSMC were transfected with 300ng in 24 well plates (c, d). Points represent individual tests; lines represent mean of n = 3 tests ± SEM * p < .05, calculated using ANOVA and posttest.

Having seen an impact of DNA architecture on expression of the SARS-CoV-2 spike protein but only marginal impact on immunogenicity, we explored another viral antigen – the hemagglutinin (HA) protein from H1N1 influenza virus. As with the other proteins, expression from dbDNA™ was quantified by transfecting cells *in vitro* and assessing surface-expressed HA via flow cytometry. In HEK293T cells, a similar pattern was seen for HA as eGFP; following F2 transfection, a significantly larger proportion of cells expressed HA than those transfected with F1 (p < .0001), Figure 4a. Likewise, MFI was greater in the F2 transfected cells than F1 (Figure 4b). A slightly different pattern was seen for HA in the HSMC. Whilst the unmodified F1 and F2 constructs led to a significantly greater proportion of transfected cells, the highest number was observed following F2D transfection (Figure 4c). The MFI reflected the pattern seen with the percentage expression (Figure 4d).

### Triple enhancer sequence improves dbDNA™ immunogenicity

**Figure 5.**
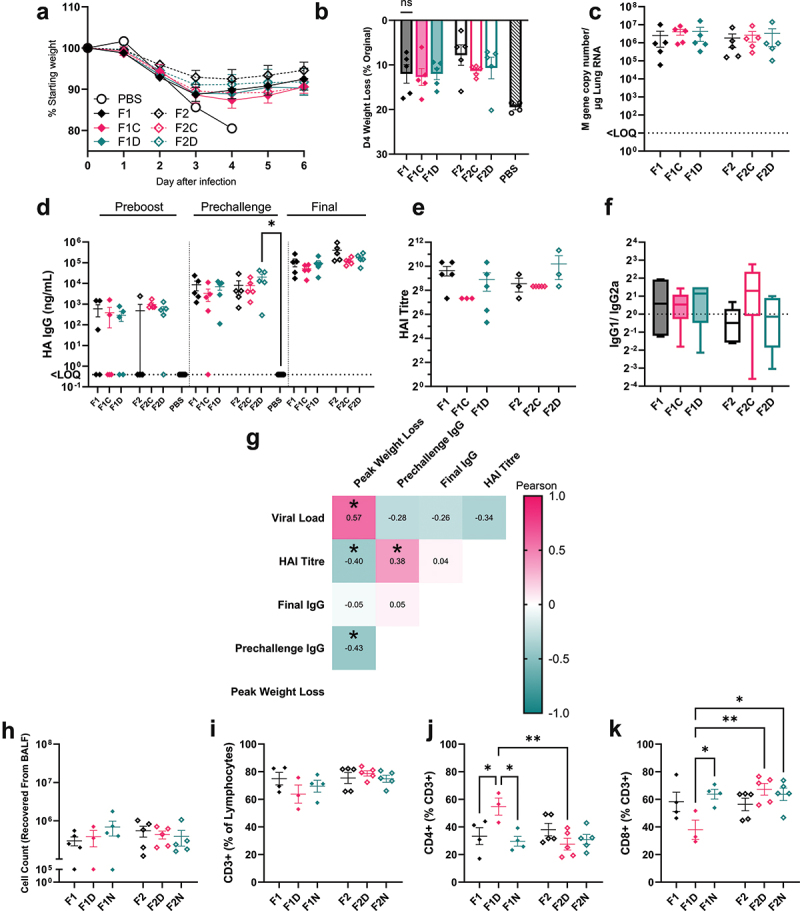
Triple Enhancer sequence improves dbDNA™ immunogenicity. Female BALB/c mice aged 8–12 weeks were vaccinated with 1 µg Cal09 HA encoding dbDNA™ in the right anterior tibialis muscle with electroporation and a second identical vaccination at 4 weeks. 8 weeks after initial vaccination mice were intranasally challenged with 25,000 PFU Cal09 flu in 100 µl of PBS. Mouse weight was monitored and recorded daily until D6 pi (a, b). Viral load was measured at experimental endpoint by qPCR of the Flu M gene (c). HA specific IgG titers were measured by ELISA of blood sera collected by tail bleed at 4 (pre-boost) and 8 weeks (pre-challenge), and by arterial bleed at study endpoint (d). Specific titers of antibody at endpoint were measured by HAI assay (e). Ratio of IgG1 to IgG2a was measured by ELISA (f). Pearson r values were computed, and results tabled into a heatmap (g). At termination, cells were recovered from the airway by Bronchoalveolar lavage (BAL): total count (H), CD3 (i), CD4 (j), CD8 (k). Points represent individual mice; lines represent mean of n ≥ 5 mice ± SEM (Data shown is representative of two experimental repeats (B F, G), * p < .05, ** p < .01, calculated using ANOVA and posttest. PBS control group was culled at D4 pi.

We then explored the effects of the inclusion of CpG or DTS sequences on the immune protection by dbDNA™ vaccines encoding HA against influenza virus infection. Mice were immunized with 1 µg of each dbDNA™ construct in a prime-boost regime with 4 weeks between injections; 4 weeks after the booster immunization, mice were infected with H1N1 influenza virus. Dose was selected to give partial protection against challenge, based on previous studies.^30^ Immunization with dbDNA^TM^ reduced influenza virus-associated weight loss and death, regardless of construct. There was a pattern of reduced weight loss in the group immunized with a vaccine featuring F2 architecture compared to the equivalent F1 architecture-vaccinated group (observed at d6; Figure 5b). There was no difference in viral load at the endpoint between all vaccinated groups, with approximately ~3x10^6^ copies of the influenza M gene per µg lung RNA (Figure 5c).

Sera were collected at 4 weeks (pre-boost), 8 weeks (pre-challenge), and at infection endpoint to assess specific IgG responses via ELISA (Figure 5d). No significant differences in antibody responses were observed after a single immunization, with many mice exhibiting IgG levels below detectable limits. At 8 weeks, F2D-vaccinated mice had the highest IgG levels, significantly exceeding those of the PBS control (p < .05). Despite an initially poor response, mice immunized with F2 had the highest IgG titers at endpoint, approximately ~3.5x greater than F1. There were no significant differences in HAI titer (Figure 5e); though F2D was higher than the other two F2 groups. There was also no difference in the IgG1/IgG2 ratio, a way to indicate T helper bias, between the vaccinated groups (Figure 5f). The primary immune correlate with viral load was antigen-specific IgG titer before challenge; viral load strongly correlated with weight loss (Pearson r statistic = 0.43, p < .05, Figure 5g). HAI titer also correlated with IgG and reduced weight loss. We also investigated T cells in the airways following infection. There was no significant difference in the number of airway cells after infection (Figure 5h) or in the proportion of CD3 cells (Figure 5i). However, there were significantly more CD4 T cells in the group immunized with F1D (Figure 5j) and significantly fewer CD8 T cells (Figure 5k). Overall, altering the dbDNA^TM^ structure had a greater impact on immunogenicity when delivering HA, in particular the F2D construct induced the most antibody – in parallel with the higher expression in HSMC.

### DNA targeting sequence in dbDNA™ vaccine reduces weight loss following influenza virus challenge

**Figure 6.**
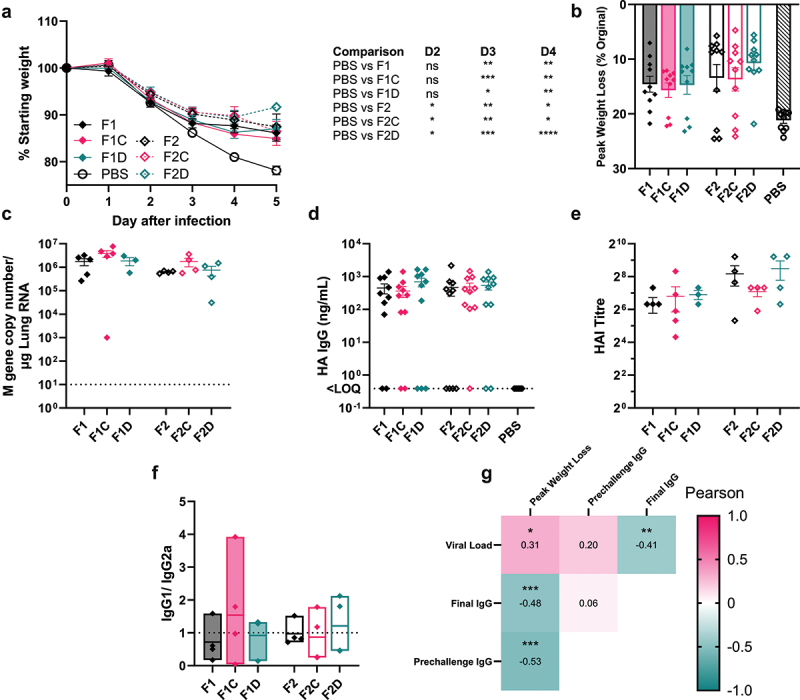
dbDNA™ with DTS better protect mice from influenza associated weight loss. Female BALB/c mice aged 8–12 weeks were vaccinated with 1 µg H1N1 HA encoding dbDNA™ in the right anterior tibialis muscle with electroporation. 4 weeks after vaccination mice were intranasally challenged with 25,000 PFU Cal09 flu in 100 µl of PBS. Mouse weight was monitored and recorded daily until D5 pi (a-c). Viral load was measured at experimental endpoint by qPCR of the Flu M gene (d). HA specific IgG titers were measured by ELISA of blood sera collected by tail bleed at 4 weeks post immunization and by arterial bleed at study endpoint (e). Specific titers of antibody at endpoint were measured by HAI assay (f). Ratio of IgG1 to IgG2a was measured by ELISA (g). Pearson r values were computed, and results tabled into a heatmap (h). Points represent individual mice; lines represent mean of n ≥ 5 mice ± SEM (Data shown is representative of two experimental repeats (B-E), * p < .05, ** p < .01, ***p < .001, calculated using ANOVA and posttest. Endpoint was either D5 or D6 post infection.

Because all groups had broadly equal levels of protection against infection in a prime-boost regime, we evaluated the effect of DTS on dbDNA™ vaccine immunogenicity/ protection from disease following a single-dose vaccination and challenge study. As seen with prime-boost, all immunized groups lost significantly less weight than the PBS control by the third day after infection (p < .05, Figure 6a). By D5 pi, the F2D immunized group had lost the least weight (Figure 6b). While a trend toward reduced viral burden was noted in mice immunized with vaccines containing the DTS, no statistically significant differences were observed (Figure 6c). Likewise, there was a trend of increased HA specific IgG in the groups immunized with architectures incorporating DTS (F1D and F2D; Figure 6d). There was also a trend to more HAI in the F2D group (Figure 6e), but no difference in IgG1/ IgG2a ratio (Figure 6f) between groups. HA specific IgG levels before infection emerged as a robust correlate against weight loss (Pearson r = 0.53; p < .0001).

### Reduced expression following co-administration of CpG ODN is compensated by increased inflammation, leading to equivalent protection against disease

**Figure 7.**
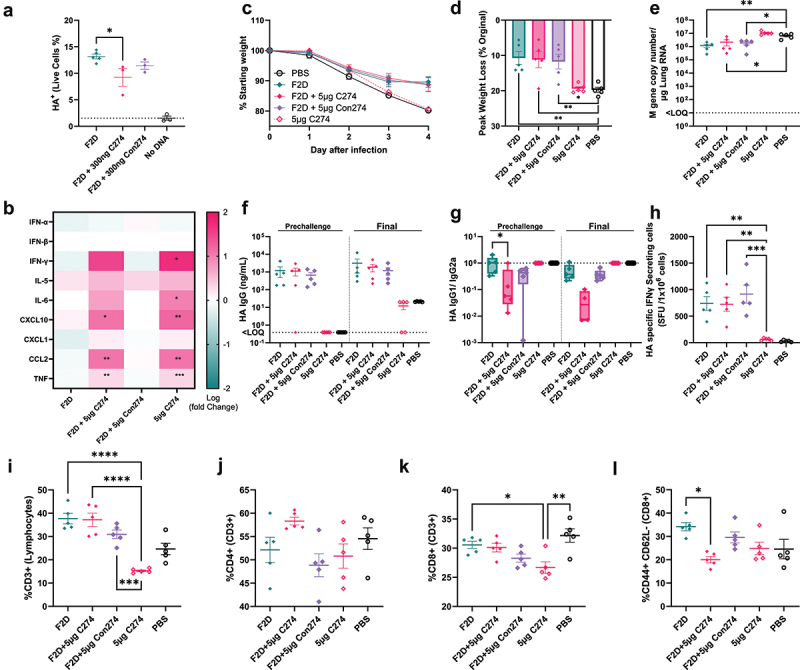
Reduced expression following co-administration of CpG ODN is compensated by increased inflammation, leading to equivalent protection. HSMC were grown to 80% confluency on 24 well culture plates. Cells were transfected with 270 ng of F2N dbDNA™ encoding HA and 30 ng of eGFP encoding dbDNA™ using the PEIpro® transfection system. Percentage of HA^+^ live cells assessed by flow cytometry. Female BALB/c mice aged 8–12 weeks were vaccinated with 1 µg H1N1 HA encoding F2N dbDNA™ in the right anterior tibialis muscle with electroporation. Vaccine was delivered alone or co administered with 5 µg of C274 or control (Con274) ODN. Control groups received 5 µg of C274 ODN without vaccination or PBS only. Systemic cytokine responses in blood sampled by venepuncture of the tail at 24 h were measured by multiplex assay (B). 4 weeks after vaccination mice were intranasally challenged with 25,000 PFU H1N1 flu in 100 µl of PBS. Mouse weight was monitored and recorded daily until D4 pi (C, D). Viral load was measured at experimental endpoint by qPCR of the Flu M gene (E). HA specific IgG titers were measured by ELISA of blood sera collected by tail bleed at 4 weeks post immunization and by arterial bleed at study endpoint (F). Ratios of IgG1 to IgG2a were measured by ELISA (G). Spleens were collected at study endpoint and the number of HA specific IFN γ secreting splenocytes were measured by ELISpot (H). At termination, cells were recovered from the lung: CD3 (I), CD4 (J), CD8 (K), CD44+/CD62L- CD8 (L). Points represent individual mice; lines represent mean of n = 5 mice ± SEM, * p < .05, ** p < .01, **** p < .0001, calculated using ANOVA and posttest.

The original hypothesis was that the inclusion of CpG motifs within the DNA construct would boost immunogenicity by stimulating the innate immune system. However, one question was the trade-off between stimulating the innate immune system and triggering cell intrinsic immunity which could inhibit expression of foreign antigens. Whilst the addition of CpG (F2C/ F1C) had a modest impact on expression in the HEK cell line which has a defect in TLR expression (Figure 4a); in the HSMC which can sense CpG we saw significantly reduced expression (Figure 4c). We therefore set out to explore whether separating the CpG from the dbDNA^TM^ would allow us to explore their different roles; based on expression levels in HSMC, we focussed on the F2D construct. HSMCs were transfected with HA encoding F2D *in vitro*, with or without the addition of CpG C274 or a control ODN (Con274 ODNs). 24 hours after transfection, the group that received 300ng C274 had significantly reduced expression of HA, by cell count (Figure 7a).

We then investigated whether there was an inflammatory effect of the addition of CpG during vaccination. Mice were immunized with 1 µg of F2D vaccine encoding influenza HA, or with PBS. F2D was administered either alone or with 5 µg of C274 ODN, an amount deliberately in excess to explore the impact. A vaccine-negative control group received C274 only. The presence of CpG C274 altered the inflammatory profile in the sera 24 hpi (Figure 7b). DNA vaccine alone or DNA vaccine plus 5 μg Con274 did not lead to a significant fold change in any of the cytokines measured compared to the PBS-immunized group. Adding C274 led to a significant increase in CXCL10, CCL2 and TNF; CpG alone also significantly increased IL-6 and IFNγ.

Four weeks after immunization, mice were infected with H1N1 influenza virus. The PBS control and CpG alone groups lost significantly more weight than the group immunized with F2D with or without 5 µg C274 (p < .05, Figure 7c, d). The vaccinated mice had significantly less viral RNA (p < .05, Figure 7e). Vaccination led to significantly greater antigen specific IgG (Figure 7f). However, the presence of C274 significantly shifted the IgG1/ IgG2a ratio, indicating a Th1 shift (Figure 7g). The addition of CpG ODN did not alter the T cell response to the vaccine, all three vaccinated groups had significantly more HA specific T cells in the spleen than control animals (Figure 7h). We investigated T cells in the lung by flow cytometry. The immunized groups had a significantly higher proportion of CD3^+^ T cells than the unimmunized control group (Figure 7i). Whilst there was no difference in the CD4 proportion (Figure 7j), there were significantly more CD8^+^ cells in the lungs of the immunized mice (Figure 7k). Interestingly the addition of C274 altered the proportion of T effector memory (TEM) CD8 cells, defined as CD44^+^/CD62L^−^; there were significantly fewer CD8 TEM in the lungs of F2D/C274 immunized mice (Figure 7L). Overall, these data suggest that the additional inflammation following the addition of CpG in trans may compensate for the reduction in expression, leading to equivalent downstream immune responses.

## Discussion

The low immunogenicity of DNA vaccines in human clinical trials has been a barrier to their wider adoption as a vaccine platform. In this study we investigated approaches to manipulate the sequence of a linear DNA vaccine platform to improve immunogenicity. We investigated two aspects of the response that DNA vaccines need to be effective; expression of the encoded antigen and inflammation to recruit immune cells to the site of immunization. One challenge is that these can pull in different directions, with recognition of the DNA leading to inflammation that recruits other cells, but also potentially supressing expression. We show that additional sequence elements designed to increase nuclear uptake enhance expression of HA protein. We also show that including CpG motifs on dbDNA™ reduces expression in vitro; these changes in vitro did not impact the downstream immune response to the vaccine. We hypothesized that the CpG, whilst dampening the expression, may be compensated by increased inflammation. To test this, we included additional CpG ODN – in vitro, this significantly reduced antigen expression; in vivo it led to higher levels of cytokines. We argue that the increased inflammation counterbalances the reduced expression, leading to an equivalent protective response. This therefore suggests that approaches that maximize both expression and inflammation will significantly improve DNA vaccines. However, reactogenicity also needs to be taken into account – increasing the inflammation may drive this up.

One of the study’s main aims was to explore the effects of sequences designed to boost protein expression in the context of dbDNA™ vaccines. One such sequence is the triple enhancer (TEnh) in the F2 family of dbDNA™. The TEnh contains the HTLV1 R, a synthetic rabbit β globin intron, and a splicing enhancer. HTLV1 R has been shown to nearly double protein expression from plasmid, and the TEnh has been previously shown to boost vaccine expression and cell-mediated immune responses in mice.^31,32^ For all of the antigens tested, HA, Spike and GFP there was some indication of increased expression when a TEnh was included (F2 constructs). In non-dividing cells, such as muscle cells, transport hindrance from the nuclear envelope prevents 90% of cytoplasmic plasmid DNA molecules from reaching the nucleus.^12,33^ Early publications reported that the SV40 enhancer DTS significantly improves protein expression from DNA immunization in murine muscle cells and enhances immune responses to HA in the context of dbDNA™ vaccines.^30,17^ Here we also identified a trend of increased HA expression from HEK293Ts following transfection with dbDNA™ with the DTS compared to that without. There was also a small, but significant, increase in HA^+^ MFI in F2D transfected cells compared to those transfected with F2, further indicating an increase in protein expression. Confirming what we have reported previously, the benefit of DTS was antigen-specific, and no benefit was found in transfection efficiency or immunogenicity of dbDNA™ encoding eGFP or Spike.^30^

The bacterial backbone of plasmid vaccines is known to induce an innate immune response, contributing to the self-adjuvant effect observed in DNA vaccines. dbDNA™ lacks a bacterial backbone, raising concerns about a potential loss of immunogenicity without these inflammatory elements. Within dbDNA™ constructs, CpG motifs are typically confined to encoded antigen and regulatory sequences. Previous studies have shown that in prime-boost regimens, TLR9 is dispensable for inducing immune responses to DNA vaccinations with either plasmid or dbDNA™, but other work has shown TLR9*^−/−^* mice have reduced survival rates when given a single vaccine dose.^34,35,36^ In this study, the inclusion of the CpG-rich region did not affect the expression of encoded proteins at 24 h in transfected HEK293T cells, which lack TLR9 expression. There was a significant reduction in HA and Spike protein expression in HSMC cells transfected with CpG containing constructs. Co-delivery of CpG ODN with dbDNA™ vaccines elicited an acute cytokine response but did not affect the level of the antibody response or protection against disease. CpG ODN did drive an IgG2 bias in vaccinated mice, as has been previously shown in other studies.^37,38^ IgG2 is a signifier of a Th1 immune response, typically characterized with the secretion of pro-inflammatory cytokines and the development of CD8^+^ T cells to clear intracellular pathogens, such as viruses. These studies indicate the need to balance inflammation and expression for nucleic acid vaccines. But more mechanistic work is required to fully understand the effect.

Further work is required to fully develop a playbook for the optimum nucleic acid vaccine. One additional challenge is that not all antigens behave the same, in this study we observed some antigen-specific effects, the mechanisms of which are unclear. Though we only tested three antigens, effects may appear across a wide range of proteins. Even membrane glycoproteins, arguably very similar in structure do not necessarily prefer the same architecture. For intron-containing sequences you can expect that mRNA stability may alter but for things like DTS or CpG, it’s less clear how these impact the transgene. The encoded antigens are different sizes; eGFP is approximately 720 bp in length, HA is 1,701 bp and the pre-fusion Spike is approximately 3,822 bp, affecting the copy number delivered in each experiment. If sequence alone affects expression, future experiments may confirm this using molarity matched dbDNA™ or by comparing antigens of similar size. Larger DNA sequences may also include more sequences recognized by innate immune receptors that can result in DNA degradation and limit protein expression. Whilst we did not look specifically here, the antigen sequence itself can have significant effects on innate sensing – for a gene the length of hemagglutinin, there are theoretically 10^200^ different possible sequences all of which encode the same protein structure. Certain antigens appear to be more immunogenic than others, possibly because of the epitopes they contain, for example we have recently observed a stronger immune response to HA than NA in the same mouse model.^30^ This study finds that the immune outcomes that occur because of the incorporation of supporting sequences are complex and dependent on encoded antigen and perhaps other sequences in the DNA architecture. The studies were performed in mice, historically DNA vaccines have underperformed in larger species, further work will be required to explore effects. Further study is required to discover if universally beneficial sequences can be designed or if vaccine architecture will need to be tailored to each target antigen.

## Biographical Note for Corresponding Author

***John Tregoning*** is currently Professor of Vaccine Immunology at Imperial College London, where he has studied the immune responses to vaccination and respiratory infection for more than 25 years. His group is currently focusing on the immune response to RNA vaccination. John has written more than 80 peer-reviewed scientific articles. He is also the author of the upcoming book *Live Forever? A Curious Scientist’s Guide to Wellness, Disease and Ageing* (due out January 2025). He previously published *Infectious: Pathogens and how we fight them* (OneWorld) which describes the history and science of infectious disease, selling over 8,000 copies.
